# Tragic Flaws and Practical Wisdom: Public reasoning behind preferences for different genetic technologies

**DOI:** 10.1177/09636625251333316

**Published:** 2025-05-09

**Authors:** Henry G.W. Dixson, Catherine Waldby, Sujatha Raman, Adrian Mackenzie, Lucy Carter

**Affiliations:** The Australian National University (ANU), The Centre of Excellence in Synthetic Biology, Australia; Western Sydney University, Australia; The Australian National University (ANU), Australia; Advanced Engineering Biology Future Science Platform, CSIRO, Australia

**Keywords:** focus groups, framing, public engagement, public understanding of science, synthetic biology, thematic analysis

## Abstract

Advanced bioengineering is often described as a transformative field with the potential to reshape aspects of society and environments. However, it remains largely unfamiliar to publics, compounded by its highly abstract and complex technical details. Increasingly, there have been calls for public engagement that grounds the field in concrete, real-world uses. Furthermore, there have been calls to move beyond the limits of archetypal or intrinsic concerns by encouraging people to flesh out and justify their support or lack thereof. This national focus group study investigated views across Australia regarding four novel applications. By presenting these technologies in contextualised scenarios incorporating characters with a range of perspectives, it answers the call for greater frame awareness. We conclude that publics are more than capable of weighing and negotiating between multiple frames at once, providing their own in order to justify whether to accept or reject one of the technologies.

## 1. Introduction

The way publics frame scientific issues can reflect their mood. A spaceflight programme could be framed as an exciting step in discovering new worlds, or a waste of resources that should be put towards more earthly issues. Synthetic biology (SynBio), or more broadly advanced engineering biology, has become a focus of public outreach aimed at better understand societal perspectives (Betten et al., 2018; Carter et al., 2022, 2023; Hobman et al., 2022a, 2022b; Macnaghten et al., 2019; Mankad et al., 2021, 2022; Steurer, 2016 [2015]; Synthetic Biology Future Science Platform (SBFSP), 2020 cf. Marcu et al., 2015; Wibeck et al., 2017). With the growth in research on public attitudes towards SynBio, there is a rising call to better understand how people frame its real-world applications (e.g. Betten et al., 2018; Carter et al., 2021), which is critical for ethical frameworks seeking to guide governance (Van Der Kley et al., 2023). Investigating the use of frames – cognitive and discursive tools that create meaning from complex issues – can help. However, little is known about how publics spontaneously frame SynBio.

Meckin and Balmer (2018) argue that devices for public conversations need to go beyond abstract future scenarios for SynBio and create ways of situating it in everyday life. Contextualised scenarios, they argue, are better able to engage publics, helping connect innovations with familiar frames of reference. Other researchers such as Ditlevsen et al. (2020) have since used specific applications to situate public and expert views. This article builds on growing interest in grounded modes of engagement, on otherwise abstract technological futures, to investigate how Australians frame real SynBio applications.

SynBio uses genetic engineering, the standardisation and modularisation of parts and high through-put construction to build and design living systems. It has the potential to address significant problems in environmental management, health, agriculture and manufacturing (Gray et al., 2018). Beyond specific, debated risks, its implementation requires a high degree of social trust as it involves intervention in areas like food production and the environment. Hence, SynBio requires social licence, which it could lose through overpromising, remaining opaque in its development and possible uses, making assumptions about society that people disagree with, or simply failing to engage publics during R&D. In other words, the way SynBio is framed could fail to resonate with people, as Betten et al. (2018) recognise.

Sensemaking, or ‘the cognitive and communicative process through which humans understand, describe, and relate to phenomena’ (Linnér and Wibeck, 2019: 11), is well served by deliberative, discursive approaches. Activities that encourage sensemaking help inform us of the thicker details regarding what people think of novel phenomena. Therefore, to responsibly develop emerging technologies within SynBio, we need to foster dialogue regarding not only how people make sense of it as a concept, but how they freely respond to the kinds of frames that are commonly found in communications, promotions and debates about possible uses. Decision making is a critical part of sensemaking (Beyer, 1981): taking an unknown and making it meaningful (Waterman, 1990), which ‘serves as a springboard into action’ (Weick et al., 2005: 409). Indeed, prior research has proven that asking participants to assume the role of government decision maker opens communication to produce lively discussions (e.g. Thomas et al., 2018). Thus, this research uses an approach that places participants in the role of hypothetical decider: which SynBio application would they roll out into society, or hold back, and why?

### Framing and SynBio

Frames emphasise the implications of something (Nisbet, 2010 cited in Bauer and Bogner, 2020). As Entman (1993) describes, framing ‘essentially involves *selection* and *salience*. To frame is to select some aspects of a perceived reality and make them more salient’ (p. 52). It goes beyond describing a technology to offering a style of thought about it (Macnaghten, 2020). It may represent exciting change, a series of risks, a moral dilemma and so on. According to Snow and Benford (1988), the success of a frame depends on how well it *diagnoses* a problem and proposes *solutions.*^1^ Diagnostic framing refers to identifying a problem and attributing blame, while prognostic framing specifies a course of action. SynBio promotions make use of both, often in tandem with governments in the form of reports and roadmaps. For instance, they might frame a given phenomenon (e.g. plastic-eating enzymes) as a breakthrough solution to a challenge we have hitherto failed to solve (e.g. non-biodegradable plastic waste). Alongside specific, proximal problem-solutions like plastic-eating enzymes, are the highly promissory, even idealistic frames around areas like energy, health and food security. These tend to coalesce into a ‘technofix imaginary’ based on enhanced control of nature (Fulvi and Wodak, 2024). SynBio is also presented as a field that, despite the unknowns, may offer a necessary gamble to address some of our biggest problems, notably climate change (cf. Fulvi and Wodak, 2023).

A key reason for any frame’s success is whether it resonates with an audience (Wibeck and Linnér, 2001; Benford and Snow, 2000; Snow and Benford, 1988). Benford and Snow (2000) break down the two main factors behind this: credibility and salience. Both, in turn, have three dimensions. Frame credibility is about legitimacy: is it perceived as empirically backed, is it consistent, and are the people communicating it trusted? Salience is about relevance to the recipient’s life on three fronts: centrality (how essential is it to the recipient?), personal experience (is it congruent with the recipient’s own experience?) and cultural resonance (does it have ‘narrative fidelity’ and connect with the recipient’s worldview?). Without these three dimensions, a frame may not resonate with an audience. However, people are not homogeneous with respect to worldviews, life experience or preferences. They will not align perfectly over who and what they perceive to be credible, nor the salience a story has to their lives. Indeed, quantitative research has shown while people view scientifically framed descriptions of gene drive as more credible, this may elicit higher risk concerns among certain segments of society, increasing polarisation (MacDonald et al., 2021). No single frame for SynBio will achieve some sort of elusive universal resonance. Therefore, investigating how people make sense of its applications requires asking them to consider several possible frames at once. This reflects more natural, real-world discourse on novel science and technology, which always involves multiple frames. It also offers participants a chance to express which frames resonate, even offering their own.

While qualitative research could improve our understanding of how publics spontaneously frame SynBio, there is very little that follows these lines. Betten et al. (2018) conducted focus group discussions in the Netherlands and showed that while people are not generally for or against SynBio, perspectives were shaped by core values regarding our relationship to science and technology, and feelings of discontent. Indeed, quantitative research shows public views on issues like climate, vaccines and gene drive are shaped by a range of social values and levels of trust in science (Dixson et al., 2023). Overall, the media’s promissory and uncritical tone regarding SynBio has contrasted with public discussions, and this shows up in framing. A content analysis of nearly 12,000 German and English-language media articles showed that religious frames were less frequent than construction and design metaphors (Braun et al., 2018). In contrast, public responses have tended to make use of narratives such as ‘playing God’ and ‘messing with nature’ (Carter et al., 2021; Dixson et al., 2022). Analysing 350 English-language newspapers, Hellsten and Nerlich (2011) showed three central metaphors when describing SynBio: organisms as books (e.g. the ‘book of life’), as machines (building/engineering new parts) and computing (e.g. programming organisms and DNA as software). However, there has been criticism of the pitfalls of framing SynBio in terms of machine metaphors (Boldt, 2016, 2018). Media framing has also made a distinction between SynBio and earlier controversies around genetic engineering. While genetic engineering was framed by end products (e.g. Frankenfoods and Terminator seeds), SynBio is framed in terms of superior control over technical processes: its continuation of the industrial revolution through the standardisation, assembly and control of parts. As the English-language media content analysis describes, this was clothed in more benign language of ‘sewing’, ‘stitching’ and ‘tinkering’ (Hellsten and Nerlich, 2011). However, as with the machine metaphor, there have been critiques of using misleadingly confident language of precision and predictability (McLeod and Nerlich, 2017), leading some to offer careful reframing of their own (Kearnes et al., 2018).

Reframing emerging technologies can involve a variety of tools. Most notably, anchoring takes unfamiliar things and relates them to something familiar. For example, Marcu et al. (2015) asked how people made sense of synthetic meat in focus group discussions. Participants anchored it to familiar issues like GMOs and used science fiction metaphors like ‘Frankenfoods’ or notions such as the aforementioned ‘tampering with nature’. As the authors discuss, people can use anchoring to go over the product or technology and express wider concerns about issues such as biotechnology in general, institutional distrust or corporate exploitation. This means anchoring does not necessarily increase desirability. Rather, it can hold the technology in an ‘unfamiliar place’, imbued with ‘risk, strangeness, and unnaturalness’ (Marcu et al., 2015: 558). Therefore, it is reasonable to expect the use of socially shared anchors during public discussions of SynBio, including metaphors, analogies and references to past technologies and issues. However, this does not necessarily mean it will render SynBio more comfortable or acceptable.

Betten et al. (2018) stress the importance of ‘frame awareness’ with respect to public discussions of SynBio, which is often communicated with a problem-solution structure. However, people may not necessarily agree with the underlying problem or opportunity. Therefore, reframing is an important part of frame awareness, whereby the problem or opportunity presented to participants is critically discussed and the focus might shift to different problems and opportunities. For example, they contrast a participant who believed SynBio will help solve global food shortages with participants who disagreed about the underlying premise that food shortages are a real problem. As the authors argue, such responses were sometimes deployed to refocus discussion on what others believed were more pressing issues. As such, people might reframe SynBio applications, and may even place the very need for them under scrutiny.

Despite the pervasive use of framing in SynBio communication, we know little about how different frames resonate with audiences (Russell et al., 2022). Frame resonance will help raise our own frame awareness by informing us as to how people spontaneously respond to a range of frames, which actors they perceive as credible, and therefore why social trust and legitimacy are won or lost. Therefore, our article investigates public responses not only to a set of SynBio applications, but several frames embedded within their descriptions. It posits that just as frames can influence people’s preferences (cf. Lichtenstein and Slovic, 2006), as per well-documented framing effects (Tversky and Kahneman, 1981), people can assess multiple frames at once and may freely choose one over the other or spontaneously generate their own based upon their beliefs and preferences. It uses open focus groups to better understand what justifications participants provided for agreeing with or rejecting certain frames, and the possible new frames they create during discussions. However, our study does not ask participants to give a definitive frame for SynBio per se. Given its myriad uses and broad range of concerns publics have regarding emerging technologies, no such frame likely exists. Instead, people were placed in the position of hypothetical decision-maker and tasked with collectively interpreting and justifying which SynBio technologies they would individually choose to roll out or hold back. We did not press for consensus in any group, allowing for individual disagreement and debate. From this, we inductively developed overarching themes that reflect spontaneously delivered public frames for SynBio.

## 2. Materials and methods

### Ethics

Ethical clearance was provided by the Australian National University’s Human Research Ethics Committee (protocol 2022/141) and a reciprocal ethical clearance process at the Social Science Human Research Ethics Committee from the Commonwealth Scientific & Industrial Research Organisation, or CSIRO (protocol 081/22).

### Sample

Recruitment was facilitated by Taverner Research Group to reduce bias and achieve a demographically representative sample. Focus group participants were recruited from the Australian public aged 18 years and older. The term synthetic biology was omitted to reduce priming participants or encouraging pre-focus group research. Instead, participants were invited to discuss an emerging scientific field and related technologies. Recruitment provided a balance of age and gender representation, using a mix of social media advertising, telephone recruitment from randomly generated landline and mobile phone numbers. Participants were recruited from across each of Australia’s capitals (state and territory) and several regions (Table 1): 57% female, average household income of 85–90k, 74% Australian born and 53% holding a tertiary degree (higher than the national level).

**Table 1.** Focus group locations.

Location	State/Territory	Participants
Canberra	Australian Capital Territory	6
Alice Springs	Northern Territory	3
Darwin		6
Regional Northern Territory Farmers		4
Brisbane	Queensland	6
Mackay		5
Hobart	Tasmania	7
Melbourne^2^	Victoria	5
Adelaide	South Australia	6
Regional South Australia		6
Perth	Western Australia	6
Regional Western Australia		4
Sydney	New South Wales	6
Regional New South Wales		4
**Total**		74

### Procedure

All focus groups took place between late-2022/early-2023. They lasted approximately 2 hours and were conducted online by two moderators (the first author and PhD candidate working in a similar area of research). They were recorded for audio and video (later transcribed through a professional transcription service). Participants first heard an expert-vetted definition of SynBio, followed by an educational video. Following initial discussions, each group was presented with two of four different possible SynBio-related applications. These were presented as vignettes, depicting hypothetical scenarios in which characters expressed highly contrasting views regarding the application. Participants then openly discussed these vignettes and were encouraged to consider whether they agreed or disagreed with the strongly held viewpoints of different characters (for further details see Supplemental Materials: Vignettes). They then had to choose which of the two applications they preferred and justified their choice. This article focuses on this closing section of the discussions relating to public reasoning around the choice of (or refusal to choose) a favourite technology to roll out into society.

The four different SynBio-related applications were:

1. Synthetic milk: a precision fermentation technique whereby cow DNA is inserted into yeast to form the same proteins found in cow’s milk.
2. Colour-gene edited cotton: colour genes are added to cotton DNA, allowing it to produce its own colour as it grows, reducing the need for dyes.
3. Plastic-eating enzymes: genetically engineered new-to-nature enzymes, which break down plastics to their core molecules for recycling.
4. Gene drive rabbits: a genome editing tool that targets and edits sections of the rabbit’s DNA, such that they only give birth to males.

### Analysis

#### Thematic analysis

This study used NVivo 14 to conduct inductive thematic analysis, adopting an exploratory, applied analytic method of constant comparison during the development of themes (Willig, 2022). Regarding theoretical assumptions, it takes a pragmatic interpretive approach whose aim is creative discovery. It is broadly in line with post-positivism: there is an objective reality, which is filtered not just through subjective but intersubjective experience. Rather than focusing on the theme maker’s subjectivity, this study emphasises how themes can be used as a tool to pursue something latent within the data—a story that exists ‘out there’ in the world of others. This is why there are recurring themes on public views of engineering nature. We see this across different studies, populations, and from analysts of different disciplinary backgrounds (e.g., Carter et al., 2021; Dixson et al., 2022; Macnaghten et al., 2019; Betten et al., 2018; Wibeck et al., 2017). The analyst plays a key role in creating and interpreting themes, but these are still grounded in participants’ words, and capable of revealing common concerns. This standpoint shaped the approach to the data, concentrating our focus on how people collectively make sense of the phenomena relating to emerging SynBio technologies, expressing latent values, preferences and understandings. This offered an opportunity to develop complementary and competing themes at different levels of abstraction. Thus, it draws from Attride-Stirling’s (2001) process for developing thematic networks. This included following a process similar to Strauss and Corbin’s (2015) 3 stage coding procedure of open, axial and selective codes. Furthermore, it advances the use of thematic networks by adding valence to thematic analysis, whereby public talk that placed technology in a negative light (unattractive/unappealing/bad) was separated from talk placing it in a positive light (attractive/appealing/good). This was done to contrast acceptance versus non-acceptance, while allowing for richer analysis of intrinsic concerns (cf. Carter et al., 2021).

#### Thematic network analysis

A thematic network (Attride-Stirling, 2001) is made up of basic themes (lower-order premises), organising themes (groups of basic themes that share some abstract principle) and a global theme (a superordinate theme that captures the principal message or metaphor of the entire text). At each stage, a more abstract level of analysis occurs. Basic themes are simple statements derived from text excerpts. Once a final, global theme is developed, the original text is now read through the structure of the thematic network, rather than in a linear fashion (for further details see Supplemental Materials: Thematic Network Process).

A coding framework was developed based on our interest in public sensemaking around emerging technologies, and on the issue of positive and negative framing. Every single sentence in the original text was coded, given the highly focused nature of the discussions (justifying the choice of one application to roll out). The focus group discussions were treated as one text, rather than dividing the analysis by location or application. This achieved the maximum coverage for overarching thematic networks, designed to broadly represent Australia. However, this necessarily removed the higher resolution of comparing geographic regions, or societal segments, against one another. Both thematic networks were re-read for their relevance and nuances regarding each of the four applications.

The data were read multiple times and dissected into segments. This produced 41 codes with a low level of abstraction, containing little meaning beyond their specific statements. They were derived from (1) the broad theoretical interest in framing and sensemaking with respect to emerging technologies, and (2) the inductive thematic analysis approach of deriving codes from the content of discussions, which in this case initially developed into themes relating to urgency, breakthrough and the dangers of intervention. Text could be included under more than 1 code and themes were developed *across* focus groups, with the intent of producing common themes.

The 41 codes were split into 2 camps: discussion around the ‘solution’ and whether it was favourable, and discussion around the ‘problem’ it was designed to help. Most codes fell into solution-based comments. Therefore, to further break this down, they were partitioned into positive versus negative qualities of the technology, which was a useful way to engage with the data in terms of framing problems, opportunities and benefits. This produced a set of positive codes and negative codes (supportive and non-supportive of the technology). These 41 codes were then refined, resulting in 17 final codes or basic themes: 9 that were positive frames (merged into 4 organising themes) and 8 negative (merged into 3 organising themes). Thus, we created two streamlined thematic networks, one positive and one negative.

We will now describe the data through the lens of these two thematic networks, one grounded in positive frames and the other grounded in negative frames. To reiterate, this discussion was in answer to the question: *If you had to choose 1 application to roll out, which would you prefer and why?*

## 3. Results

### Initial impressions

Due to the nature of the applications being considered, discussions revolved around the environment and pollution. Hovering over this were concerns about over-controlling nature, the loss of control due to unnecessarily hasty decisions, and the wastefulness of consumerism. Simultaneously, much of the content involved expressions of agreement about the need to act definitively on various environmental problems, and sympathy towards the use of SynBio. Although this was not a quantitative study, more participants preferred plastic-eating-enzymes than any other option, and this application engendered less negative talk. We will now present and describe the two thematic networks that summarise the data set. The first network was developed from frames that placed technology, or the subject matter around its discussion, in a negative light. The second network was developed from positive frames.

### Tragic Flaws

**Figure 1.**
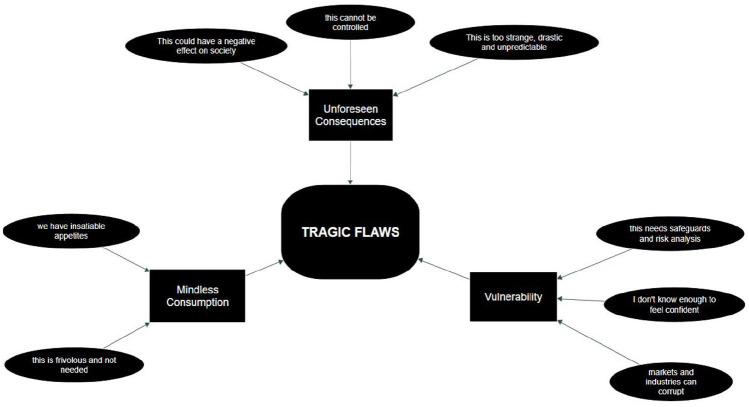
Negative thematic network: ‘Tragic Flaws’.

The thematic network, Tragic Flaws, tells the story of mostly negative talk across focus group discussions. Although this was predominantly a discussion about the four SynBio applications, talk included related topics like decision making, society and human behaviour. The global theme in the centre (rounded rectangle: Tragic Flaws) encapsulates the three lower level organising themes (rectangles: Unforeseen Consequences, Mindless Consumption, Vulnerability), which ultimately capture the meaning of the very low-level-abstraction basic themes (ellipses), based on clusters of individual text segments (Figure 1). Each application stimulated concerns about the loss of control side-by-side more positive frames described in the next network. We begin with the first organising theme: Unforeseen Consequences.

#### Unforeseen consequences

Like on the surface it sounds ok . . . In my heart, I’m just not 100% convinced we know all the implications for down the track . . . Like feral cats in Australia . . . I don’t mind if they get Aboriginal people to track them down and kill them . . . I know that’s violent as well, but it’s short-term stuff and long-term DNA modifications, I don’t fully understand, (I) feel nervous about that. (Alice Springs)

Rather than *unintended consequences*, analysis developed the key theme of consequences that were unforeseen. There is an important distinction here. While the common label unintended consequences refers to negative outcomes that were not supposed to happen, unforeseen consequences are negative outcomes that were not anticipated or could not have been known due to their complexity (unknown unknowns). This is a common concern regarding changes to DNA, as expressed in this exchange about gene drive rabbits in Alice Springs:

D: ‘I don’t know we can be sure (about) the long-term implications of mucking around with rabbit DNA’.

L: ‘I’d go with synthetic milk because I think the rabbit business is really drastic and heaven knows where that would end’.

G: ‘Environmentally, I think they’re both coming from sort of good places . . . but I think interfering with genes is a bit too drastic for me’.

This raised the contradiction of why some were willing to accept one form of genetic engineering (synthetic milk) over the other. This was justified by the fact that rabbits are not inert, by the fact that they reproduce, and by their ontological status as animals rather than a chemical substance or plant. Therefore, the tools of SynBio were reframed as inherently risky to the environment when applied to animals, but *less* problematic with respect to chemicals, substances and plants. As these participants from Canberra describe,

A: ‘I’d go with cotton. Sounds safer’.

M: ‘Yeah, I feel the same about cotton. I think it’s got a very positive impact on things but it can’t be tampered with too much. Whereas the other one (gene drive rabbits), we don’t know where it’s going to end up or where or what it’s going to lead to’.

In earlier discussions delving into initial reactions to gene drive (not reported here), prior biosecurity errors such as cane toads and myxomatosis featured quite heavily. In our current analysis, one farmer from the Northern Territory used strong imagery including the loss of controllable boundaries, and blending themes of airborne disease with being overrun by rabbits, which carries weight in Australian agriculture:

Look, I’m not sure how I feel about that. It’s a bit like your neighbour using chemicals and coming across your boundary fence . . . I don’t like the fact that someone else’s decision could impact our enterprise. You let something go, like a mosquito that’s going to spread this issue with the rabbit – there’s no controlling that. It’ll go from one end of Australia into the other. If it finds an area where there’s no rabbits it could find something else to infect. So, who knows, unless you test on every single animal in Australia or in the world, where is it going to stop?

Gene drive provided most of the content for Unforeseen Consequences. However, as will be illustrated in the positive thematic network, it also inspired a lot of talk regarding its special importance to the Australian context. Furthermore, other applications were part of this theme, but to a lesser extent. Notably, synthetic milk inspired health concerns regarding taking something into the body that was uncanny and ‘weird’ (South Australia), whereas colour-gene-edited cotton was ‘going on our body, not in our body’ (Western Australia). Frequently, participants expressed a preference for neither, but if forced would choose based upon lower health or environmental risks. Perhaps the most intriguing depiction of this choice as a lesser of two evils came from an Aboriginal-descendant participant from Western Australia:

I prefer the milk because of the water situation. So, cotton really damages our – well to my opinion, cotton develops our water cycle – damages our water cycle. So, it’s a spiritual problem . . . but the milk thing . . . that’s just a system and structural problem. So, it’s a lesser of two evils, the milk I think, for me.

Here, two forms of harm are not equal because of two qualitatively different systems: one natural and spiritual, and the other a human made, or profane structure. This places them under a moral hierarchy. The natural world and its biogeochemical cycles, such as water, are a spiritual problem when *developed* by society, while markets and sectors, such as dairy and agriculture, are human structures, and therefore less damaging when subjected to our interventions. Although other participants did not clarify their responses under such terms, this ontological framework succeeds in exposing the heart of the organising theme Unforeseen Consequences: natural and human made systems are already complex, but the former ultimately undergirds the latter and they interact in such a manner that complexity and uncertainty are compounded. Thus, overturning this nature-society relationship will lead to cascading effects throughout both natural and societal systems. This theme had the highest amount of content of any theme, and should not be dismissed as low information ignorance, a pathological lack of trust, or irrationality.^3^

#### Mindless consumption

The organising theme Mindless Consumption frames human activities as often wasteful and vain. Most of the content for this theme came from responses to colour-gene-edited cotton. Although SynBio tools are being developed for a variety of environmental purposes with respect to cotton, as described earlier the vignette chose colour-gene-edited cotton, and included the frame that this application was good for the environment as it helped reduce the development and use of industrial dyes. Indeed, this vignette intended to make clear this benefit, and to do so in a manner that was accessible and relatable. However, many participants reframed this as an aesthetic concern, often voicing opposition to prioritising this over other issues. Therefore, while colour-gene-edited cotton had an advantage over gene drive rabbits or synthetic milk in terms of safety – more containable and something that goes ‘on the body, not inside the body’ – it was often associated with the human flaws that created environmental problems in the first place. Thus, it could increase wasteful consumption because of ‘novelty value’ (Adelaide):

It’s purple in this field and it’s red in that field and it’s green in that field. Oh no, I don’t want that shade of green; I want this shade of green. This shade of green’s out this year. It’s lime green this year . . . I can see a benefit but compared to (gene drive rabbits) . . . Not even a choice. (Brisbane)

So, plastic, plastic, plastic. Find a way to get rid of it. Who gives a shit about the colour of somebody’s dress? Wear a white one, it’s still just exactly the same. (Darwin)

This reframing shifted the original pro-SynBio application frame from the environment and reset it beneath the problem of human consumerism. This also occurred with synthetic milk. Frequently, talk moved to new milk alternatives as unnecessary, both in light of pre-existing options and the very consumption of milk:

I don’t think there’s a need for it. If milk never existed, goats’ milk, almond milk, other milks. There’s a million oat milks, there’s a million milks out there. If cows’ milk never existed, I don’t think that’s a big loss personally. (Mackay)

In short, participants sometimes rejected the frame. Human decisions were framed as frequently wasteful and weird. Humans are, for instance, the ‘only animal in the world, other than cats, that drink milk after they’ve been weaned’ (South Australia). Therefore, this theme was as informed by what it *didn’t* feature than what it featured: there was a distinct lack of content on plastic-eating-enzymes and gene-drive-rabbits (except for some discussion around encouraging more consumerism through converting plastic waste back into plastics). This is because they achieved a greater level of frame resonance, despite concerns around safety.

#### Vulnerability

Vulnerability was the final organising theme of Tragic Flaws. This theme emphasised the need for careful safeguards and risk analysis, the possibility of industrial exploitation and low confidence in the subject matter due to its technical complexity. Often, this was tied with an emphasis on environmental and regulatory protections. As one participant described,

I’d need more information regarding how to make plastic-eating-enzymes safe, both for people, for our community, for the fact that it’s great they eat plastics, but we don’t want them to eat the wrong plastics. (New South Wales)

One of the reasons for a need for appropriate regulation related to poor self-regulation among companies. This underscored the vulnerabilities of people and environments to novel, transformative technologies such as a plastic-eating-enzymes. The feature distinguishing Vulnerability from Unforeseen Consequences was its emphasis on *anticipating known problems* and concern that these would not be heeded. As one participant from Alice Springs explained,

I would just hope that caution was shown, and they did lots of testing. I think it’s worth following through on the idea (plastic-eating-enzymes) with full testing and safety.

Therefore, of the three organising themes, Vulnerability was the least negative and most constructive in terms of supporting SynBio applications. Nonetheless, the theme captures a cautious tone around financial interests, a strong need for regulation, and our inherent vulnerability to a lack of safeguards and clear information.

#### Summary of negative thematic network: Tragic Flaws

The thematic network ‘Tragic Flaws’ was characterised by three core concerns. The first moved beyond unintended consequences, highlighting outcomes that are unknowable due to the intrinsic unknowability of intervening with complex, nonlinear systems (see Fulvi and Wodak, 2023), whether ecosystems, populations or society. This concern regarding the cascading effects of releasing genetically modified organisms or biological parts is reflected in public discussions on the use of gene drive techniques for conservation in New Zealand, whereby the complexity and unpredictability of DNA is compounded due to shifting ecosystems and dynamic evolutionary processes (Dixson et al., 2022). However, like the argument in Fulvi and Wodak (2023), these results from Australia did not mean participants were unanimously averse to SynBio (although some were); rather, discussions emphasised how we should be clear on the extent to which its tools, when delivered, represent an unknown-unknown. The second core concern was a critique of humanity’s tendency to be vain and wasteful. On the face of it, this could serve SynBio promissory narratives about fixing environmental problems. However, our discussions sometimes re-framed tools as primarily an aesthetic choice, and our desires for such applications as insatiable hunger for more products, rather than a sustainable system per se. Finally, vulnerability expanded concerns to foreseeable societal consequences such as industrial exploitation, the corruptibility of markets, and a strong emphasis on regulation. Thus, these three core concerns informed the global theme. This global theme encapsulated talk around why these SynBio applications were either rejected or placed in the balance, a critique that transcended the field to label a quality cutting across life, society and human desires: Tragic Flaws.

### Phronesis (Practical Wisdom)

**Figure 2.**
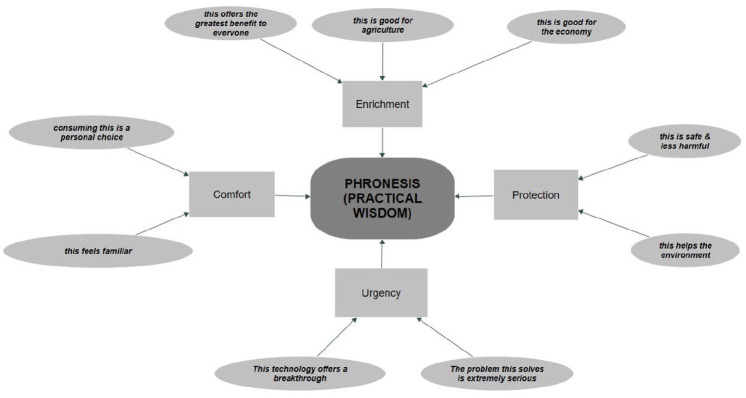
Positive thematic network: ‘Phronesis: Practical Wisdom’.

The thematic network, Phronesis (Practical Wisdom), tells the story of mostly positive talk across focus group discussions. The global theme in the centre (rounded rectangle: Phronesis (Practical Wisdom)) encapsulates the four lower level organising themes (rectangles: Enrichment, Comfort, Urgency, Protection). Again, these organising themes ultimately capture the meaning of the basic themes (ellipses), built from clusters of individual text segments (Figure 2).^4^ Each application engendered talk about considerable benefits to the environment, the economy and humanity *writ large.* Related positive themes involved anchoring the technologies in the familiar, or in preferring one over the other because it was perceived as less harmful. Finally, this part of the discussions agreed with the core framing of SynBio tools: they offer a breakthrough in solving extremely serious, recalcitrant challenges. We begin with the first organising theme: Enrichment.

#### Enrichment

Across focus group discussions, the SynBio applications were described as providing multiple benefits across society. This parallels the idea, often repeated in policy circles, of co-benefits. Although plastic-eating-enzymes were a strong feature of this theme due to perceptions of stronger impact on the world stage, each technology was present to some degree. As one participant from Brisbane explained,

Am I only to pick one? Because, realistically, I would go for both. If I had to choose one, it would probably be (gene drive rabbit) . . . The benefits to the agricultural industry would be immense and more importantly it’s going to be creating so much more improvements down the road in how we manage pest control . . . It would be really revolutionary.

There was some overlap between Enrichment and Urgency in the form of references to a breakthrough. However, with Enrichment breakthrough referred to co-benefits (as per the above quote). For Urgency, breakthrough was framed in terms of a solution to dire, unsolved problems. Indeed, another comment from Brisbane captured the appreciation for SynBio as a field with extensive potential, despite the kind of ambivalence shown in the negative thematic network:

I’m very on the fence about all of these technologies because I don’t understand them, but I do understand the important cultural . . . the agricultural and economic and just overall biodiversity (implications for) our country.

While plastic-eating-enzymes and gene drive rabbits were spontaneously framed as helping to revolutionise environmental problems (thus, showing frame resonance), synthetic milk and colour-gene-edited cotton tended to be reframed as less dangerous: providing *some* benefit with less risk. In other words, the core framing of cotton and milk – addressing environmental pollution (cotton) and protein deficits and farming pressures (milk) – did not resonate with our participants. On the contrary, as we have found in other parts of discussions that are not the focus of this article, with respect to milk there was a degree of sympathy and support for farmers. Therefore, Enrichment describes the capacity for SynBio applications to provide benefits to pre-existing sectors, while Protection (described later) leans into its potential to reduce the damage of certain sectors.

#### Comfort

Comfort was a relatively smaller theme. However, it was developed given that its content did not readily fit the other organising themes, as well as the important role that anchoring can play in sensemaking and framing. Here, participants enthused over the conceptual familiarity of an application. Strangeness is a common response to SynBio and other emerging fields. But by appearing somewhat quotidian, certain applications were less uncanny. Colour-gene-edited cotton featured strongly here, and while people expressed discomfort over consuming synthetic milk, they simultaneously expressed confidence in its relationship to personal agency. As one farmer from the Northern Territory explained,

I’d probably go with the synthetic milk product because . . . it’s not like they’re saying from now on you will only be able to buy synthetic milk. People still have a choice around if they want to buy into that product or not, whereas the breeding only male rabbits, that seems like a lot more of a drastic change.

It is telling that this was a common sentiment among dairy farmers. Rather than a change to the status of ecosystems brought about by gene drive organisms, which they (and, ultimately, everyone) cannot opt out of, synthetic milk was something over which people could make a mundane personal choice. However, its association with agency did not necessarily mean people want to consume it. As one participant from South Australia said, echoing the sentiment of other participants: ‘(Synthetic) milk? No, not in my body. No’. Regardless of the conclusions drawn, the theme was rooted in the same principle of greater ease due to familiarity with, and personal control over, the application’s effect on their life.

#### Urgency

Participants often framed plastic-eating-enzymes, and to a lesser extent gene drive rabbits, as possible breakthroughs in particularly environmental crises. Thus, these two applications enjoyed a higher degree of frame resonance. Despite concerns regarding control, the problem of plastic pollution required a breakthrough that this technology finally permits, as the following quotes from an exchange in Adelaide depicts:

S: I think there’s more of a need for that than perhaps engineering cotton that can be pink and so on. The reality is that plastic is polluting our oceans, killing . . . a multitude of wildlife. So, to me, there’s probably more urgency . . .

J: Yeah . . . I’d definitely rollout the plastic-eating-enzymes first. Mainly because that could have an immediate effect now. . . the plastic things could actually prevent pollution going into our oceans. It could also be used to recycle rather than have to keep finding more fossil fuels . . .

S2: I think the general public would be more behind the plastic-eating-enzymes and therefore it would have more chance of success and of perhaps opening our minds to being more positive about other types of synthetic (biology) technology.

Therefore, as described earlier, while Urgency shares some overlap with Enrichment by emphasising the breakthrough potential of the application (predominantly plastic-eating-enzymes), this breakthrough was based on solving a recalcitrant problem rather than providing co-benefits.

#### Protection

Finally, Protection is the last organising theme in the positive thematic network. However, while some of the content revolved around protecting the environment, most of it was based on choosing the application that was perceived as *less dangerous*. Here, as with the organising theme Comfort, colour-gene-edited cotton featured more strongly than in other themes. This exchange from Canberra captures this aspect of the theme:

A: I’d go with cotton. It just sounds . . . safer.

M: Yeah, I feel that way about the cotton. I think it’s got a very positive impact on things, but it can’t be tampered with too much. Whereas the other one, we don’t know where it’s going to end up or where or what it’s going to lead to.

Gene drive rabbits and plastic-eating-enzymes were sometimes associated with a catastrophic loss of control and a lack of clarification around how they would be controlled outside of lab conditions. In contrast, colour-gene-edited cotton was framed as inherently less dangerous as it is inert and is not internally consumed, as with synthetic milk. Its relative absence in the prior negative thematic network’s theme Unforeseen Consequences also warrants mentioning: gene drive in particular raised serious concerns about unanticipated, cascading effects, while colour-gene-edited cotton was received as relatively benign. Therefore, this final theme of the positive thematic network focuses not merely on comfort with the technology’s familiarity, but in the low risk it poses to people and ecosystems, which require multiple strategies to protect. These two themes – Comfort and Protection – were based more strongly in the qualities of an application not to *worsen* problems rather than fix things per se. Their inclusion in this network is particularly important in an Australian context, given Australia’s history of biosecurity controversies.

#### Summary of positive thematic network: Phronesis (Practical Wisdom)

The thematic network ‘Phronesis: Practical Wisdom’ was characterised by four core concerns: co-benefits, familiarity, breakthrough and self-protection. This network contained perspectives that overlap to a degree with Betten et al.’s (2018) categorisation of public SynBio views that were either ‘instrumentalist’ (risk-opportunity discourse and the benefits of SynBio) or ‘adaptivist’ (flexibility in a world of change and emerging technologies). However, we found two distinct frames were deployed to justify why a SynBio application was desirable or fit for purpose: familiarity and breakthrough. While participants were often curious and optimistic about the range of SynBio’s applications across different sectors, there were persistent themes around the need for a degree of comfort regarding something outlandish and unfamiliar (i.e. genetic engineering). In this regard, concrete SynBio applications functioned to anchor this complex, often jarring field in everyday uses and products. Some of these products (e.g. cotton) were more likely to be viewed as lower risk due to its everyday use and the fact that it was inert, not internally consumed, or released into a complex ecosystem. However, one of the strongest reframes offered across these discussions was the need for breakthrough technological solutions to crises. These two competing frames (the safer nature of lower risk, familiar products and the urgent need for breakthrough) were not evenly represented across applications: familiarity related mostly to colour-gene-edited cotton (and to a lesser extent to synthetic milk) and breakthrough to plastic-eating-enzymes and, to a lesser extent, gene drive for pest suppression. This speaks to a plurality of perspectives regarding acceptance of SynBio: it is not merely a matter of agreeing to its wholesale use or ban. Therefore, four core themes informed the global theme around why these SynBio applications were either acceptable or overtly encouraged. They reflect a pragmatic ethos with social concern: Phronesis or Practical Wisdom.

## 4. Discussion

This study offered an in-depth analysis of public sensemaking regarding concrete SynBio applications, moving away from the abstractions of the overall field and anchoring it in specific social and environmental uses. By prodding participants to choose an application and justify its roll out or refusal to do so, our findings illustrate both maturity and range in how publics assess SynBio applications. The two thematic networks – Tragic Flaws and Practical Wisdom – reflect well-established intrinsic concerns as well as a pragmatic attitude regarding tough challenges.

Although we did not set out to deliver the silver bullet for framing SynBio, this study highlights the flexibility publics can show regarding a range of possible frames. People are not as rigid as commonly assumed (especially by SynBio researchers), due in part to the familiarity of certain applications, as well as the complementary roles of urgency and breakthrough. For example, certain applications (e.g. plastic-eating-enzymes) were spontaneously framed by participants as containing risks that were eclipsed by the ever-growing issue they were framed as addressing (in this case, global plastic waste, including oceanic). It was not that technologies like plastic-eating-enzymes didn’t inspire ‘fears’, but the urgency and intractability of the wider issue transcended these. This spontaneous public framing provides a different perspective on intrinsic concerns: the role of dynamic scenarios involving urgency and our failure to resolve something. This dynamic does not remove public questions and even pushback stemming from moral boundaries about the unintended consequences of advanced genetic engineering tools. However, it does suggest there is flexibility to intrinsic concerns when discussing emerging technologies: intrinsic concerns force the conversation to acknowledge unknowns but they can also push it to clarify a need for change. In these discussions, this took the form of the severe conditions under which transformative technologies might now be needed, despite their uncertainties. Therefore, this work develops the role of archetypal narratives or intrinsic concerns (see Dixson, 2025; Carter et al., 2021; Van der Burg, 2016) by adding to the growing body of qualitative research demonstrating public attitudes can be well-reasoned and accepting of uncertainty (e.g. Kirk et al., 2020). Providing valuable information for science communicators to consider the frames used in stories, these findings should help further dissuade SynBio practitioners from solely relying on persuasive communication devices to improve public science literacy.

There are multiple roles and values of pursuing open dialogue with publics during engineering biology R&D. The normative function of transparent and substantive exchange between scientists and publics is well formulated (Carter et al., 2023; De Graeff et al., 2019; Gray et al., 2018), and is the preferred type of engagement (National Academies of Sciences, Engineering, and Medicine (NASEM), 2016). There are also important informational roles that dialogue can play in the public reception of science. Carefully planned dialogue offers an opportunity for scientists to learn about societal views while publics are provided with the latest information on advances in science. The learning, when done right, can be mutually advantageous.

### Limitations

This research purposefully made use of various frames embedded in vignettes about different applications. However, this presents a particular limitation: the need to formally operationalise frames across vignettes. Future qualitative research that presents publics with naturalistic vignettes such as ours would benefit from formalising pre-established frames (e.g. economic vs religious vs personal agency frames) such that each vignette has an equal proportion, to avoid biasing discussions towards a single frame. A further limitation of this study was the number and nature of applications. Due to time constraints, we opted for four technologies (two per focus group), with each having relevance to environmental uses. However, SynBio cuts across industries, including energy, manufacturing and health. Furthermore, the preliminary framing used in the description of each technology could easily have leaned into a different logic (e.g. economic). Therefore, future research would benefit from presenting a wider range of products and applications. Finally, this study focused broadly on Australian views. However, SynBio is increasingly recognised as needing ethical frameworks and governance strategies capable of functioning across the culturally diverse Asia Pacific region (Van Der Kley et al., 2023) and the globe (Betten et al., 2018). Advanced genetic research and development is occurring across the world, and its effects, positive and negative, are unlikely to be uniformly spread. Considering highly contrasting societal values, economic implications and political structures, public engagement in this space needs to broaden its cultural scope.

## 5. Conclusion

This national focus group study investigated spontaneous public responses regarding a set of expert-vetted SynBio applications. We presented participants with scenarios and asked them to justify which application they would roll out into society or withhold from it. We found that people could hold a range of competing frames around the acceptability of advanced genetic engineering tools, negotiating their respective merits. These results suggest people do not merely respond to the coercive quality of frames, measured by the extent to which they support one over another, but consider diverse frames at the same time and even spontaneously develop their own to create meaning and justify action: their decision to ‘release’ the product. Therefore, this study has attempted to answer two calls: to move beyond archetypal stories in emerging technologies (e.g. ‘Playing God’ and ‘Messing with Nature’) by provoking participants to reflect on specific details and justify their choices (Van der Burg, 2016); and for greater frame awareness in public views on SynBio, notably through perception-shifting opportunities for public reframing (Betten et al., 2018). Our study shows that, given the opportunity, people will do exactly that. Future research should recruit participants across a greater range of cultural, religious and political backgrounds, include non-environmental frames and a larger number of applications.

## Supplemental Material

sj-docx-1-pus-10.1177_09636625251333316 – Supplemental material for Tragic Flaws and Practical Wisdom: Public reasoning behind preferences for different genetic technologies Supplemental material, sj-docx-1-pus-10.1177_09636625251333316 for Tragic Flaws and Practical Wisdom: Public reasoning behind preferences for different genetic technologies by Henry G.W. Dixson, Catherine Waldby, Sujatha Raman, Adrian Mackenzie and Lucy Carter in Public Understanding of Science

## Notes

1. As their work bases this in the context of social movements, they present a further feature of how well a frame offers a ‘call to arms’ for mobilising people to collective action.

2. The Melbourne focus group was not formally asked the ‘roll out’ question due to time limitations. Therefore, Melbourne constituted only a small contribution to the data analysis.

3. Here, *irrational* is used rhetorically to label something as incorrect and absurd, rather than in the psychoanalytic sense (cf. Fritzsche, 1995).

4. The titles of these basic themes are not direct quotes; they act as a statement intending to capture text segments coded to this theme.
