# Supplementary material for: Tragic Flaws and Practical Wisdom: Public reasoning behind preferences for different genetic technologies
Source: Public Underst Sci. 2025 May 9;34(8):1009–27. doi: 10.1177/09636625251333316 (PMC12535613; doi:10.1177/09636625251333316)
Supplement: sj-docx-1-pus-10.1177_09636625251333316 – Supplemental material for Tragic Flaws and Practical Wisdom: Public reasoning behind preferences for different genetic technologies [file sj-docx-1-pus-10.1177_09636625251333316.docx]

**SUPPLEMENTAL MATERIALS**

Tragic Flaws & Practical Wisdom: Public Reasoning Behind Preferences for Different Genetic Technologies

Authors: Henry G. W. Dixson, Catherine Waldby, Sujatha Raman, Adrian Mackenzie, Lucy Carter

CONTENTS

**Section Page**

Thematic Network Process 2-4

Focus Groups Protocol 5-7

Vignettes 8-12

**Thematic Network Process**

Given the idiosyncratic nature of thematic analysis, details of procedures are best provided at each point of analysis (c.f. Levitt et al. 2018). We used an applied analytic approach (Willig, 1999), which was exploratory in nature. Data familiarisation and early coding was done via constant comparative content analysis (a method based on grounded theory), which followed Corbin & Strauss’s (2015) three-step coding procedure of open, axial, and selective coding. The first author read through the text of 14 focus groups, developing themes according to the interests of the study. This inductive process involved iteratively reading the raw transcripts and coding. During this early phase, notes were taken including potential higher-order themes and other relevant observations.

*Preliminary codes*

An initial analysis produced 28 preliminary codes. However, these 28 codes were only treated as an initial sweep for familiarisation: they were a mix of basic and more abstract codes. Further, many only had one or two segments of text, and were two vague. We decided that a more thorough, consistent thematic analysis required producing only basic codes at the start, and gradually working these up to more abstract themes as per the methods described in the manuscript.

The 28 codes: *Pollution & Environment; Frivolous Consumption (and a counter theme, simply labelled ‘counter’ which disagreed with the tools as frivolous consumption); Energy; Huge Problems; Costs vs. Benefits; Need More Information; Risk; Control & Leaks (and a counter theme labelled ‘enhanced control’); Market Dynamics; Breakthrough; Corporate Problems; Research; Other Options; Cannot Decide; Extinction & Cruelty; Past Catastrophes; GM is Normal; Health & the Body; Weird & Unnatural; Personal Choice; Dangerous DNA; No Limits; Safeguards; Farmers; Miscellaneous.*

These above 28 codes were treated only as a first sweep and part of data familiarization. After this first sweep was conducted, a second, more thorough code development occurred. This produced *41 codes.* Many of these codes overlapped/were redundant, or were overly abstract and needed removing (e.g., “fear of the unknown” would be removed for more specific, lower abstraction fears grounded directly in text, e.g., “could there be a lab leak?”, “what are the unseen health effects?”, etc). This was based on common discourse: themes across focus groups rather than themes specific to any single group.

To break down this list of 41 codes, as there were too many and there was redundancy, the first author split most of them into two broad categories: talk of the *Problem* (e.g. invasive pests or plastic pollution) and talk of the *Solution* (reducing invasives through gene drive or cleaning up plastic waste with enzymes). Most codes fell into solution-based comments, or comments relating to the technology and the nature of this proposed solution. Therefore, to help break down the solution-based comments further, these were partitioned into *Positive* qualities (e.g., the enzymes solve a serious problem quickly) and *Negative* (e.g., this might encourage more plastic consumption in society). We found this to be a particularly useful way to engage with the data around or core theoretical interest – *framing.* In this case, this was framing problems, opportunities, benefits, risks, etc. This also permitted new methodological developments around Attride-Stirling’s ‘thematic networks’: a *Positive* and *Negative* thematic network. This developed inductively over the course of analysis and not deductively.

These 41 codes were then partitioned into the two groups, *Positive* and *Negative,* for an iterative stage of separate analysis: both groups were separately combed over and refined to 17 codes, separated into 9 for the *Positive* group and 8 for the *Negative* group. Another phase of thematic analysis now occurred *within* each group (i.e., not comparing codes across the two networks). This strengthened the networks as two distinct stories.

During this phase, redundant codes (e.g., "fear of genetic modification" and "discomfort with genetic modification") were merged due to overlapping meaning, while others were deleted to ensure conceptual clarity and coherence. This iterative refining process helped distil those most salient 17 basic themes for the final analysis, which survive in the final thematic networks in the manuscript (the outer-most themes depicted in each network). However, as is common in qualitative research, we did not maintain a detailed record of the prior code iteration (i.e., those preliminary 41 codes, many of which were deleted or merged into the basic themes that made up the two published networks).

*Networks*

These two groups of codes were developed into thematic networks. The thematic network process followed the method of taking lower level, or less abstract, codes and clustering them into more abstract themes. This occurred in two levels: the most basic, least abstract codes called basic themes were clustered into a more abstract organizing theme, which had a moderate degree of abstraction. Then, organizing themes were read together for potential converging thematic relevance (this necessarily required a higher level of abstraction to capture the broader meaning the organising themes).

As described, in this analysis, once very low abstraction codes (basic themes) were partitioned into to two initial groups (a *Positive* and a *Negative* group), the groups were read separately, to help cluster their respective basic themes into organising themes. These organising themes were further refined through re-reading the core transcripts. Then, a superordinate or global theme was developed for each (the *Positive* and *Negative* network), which captured the sentiment of the text in both groups. This resulted in two separately developed thematic networks, each with a main global theme, organising themes stretching out from that, and the lowest-level basic themes (simple statements, questions, comments from participants) beyond those.

This is best illustrated with some examples:

Example 1: *Unforeseen Consequences (from the negative thematic network)*

The organizing theme “Unforeseen Consequences” was developed from a group of less abstract, basic themes drawn directly from the data. Here are two examples of text segments that contribute to the more abstract organising theme:

*Quote 1:* “I don’t know that we can be sure (about) the long-term implications of mucking around with rabbit DNA” (Adelaide).

*Quote 2: “*In my heart, I’m just not 100% convinced we know all the implications for down the track… I know it’s not violent as well, but it’s…long term DNA modifications I don’t fully understand, feel nervous about,” (Alice Springs).

Organizing theme: *Unforeseen Consequences.*

We deemed these kinds of statements to “belong together” under the more abstract concern – dangers of unforeseen consequences. These consequences may occur from advanced engineering biology applications acting outside of the lab by: (1) damaging society, (2) being uncontrollable once released, and (3) being too strange and drastic as a go-to method for society to take on. Therefore, the higher-order theme, *Unforeseen Consequences*, represents a cluster of these 3 lower-level themes. The increased abstraction was based on looking for principles that encapsulates lower-level themes. Here, we looked for overarching, moral messages that captured the concerns expressed in basic themes.

Example 2: *Enrichment (from the positive thematic network)*

The organizing theme “name” was developed from 2 less abstract, basic themes drawn directly from the data: “name the basics themes”. Here are two examples of text segments that contribute to the more abstract organising theme:

*Quote 1:* “This (gene drive) has got more ramifications and more environmental

factors. It’s just more beneficial, strong point for the overall environment and farmers,” (Brisbane).

*Quote 2:* “It (SynBio milk) has the potential to revolutionize the dairy industry and have all those flow-on benefits if it’s also found to be safe,” (Hobart).

Organizing theme: *Enrichment.*

We deemed these kinds of statements to “belong together” under the more abstract concern – Enrichment. These benefits may occur from advanced engineering biology applications improving various aspects of society: (1) the economy, (2) agriculture, and (3) humanity and society overall. Therefore, the higher-order theme, *Enrichment*, represents a cluster of these 3 lower-level themes. As with *Unforeseen Consequences* above, the increased abstraction was based on looking for principles that encapsulates lower-level themes. Again, both groups required developing a set of organising themes like these, clustering them together, and developing one main, global theme.

**Focus Group Protocol**

**Focus Group Discussion Materials**

Public Views on Emerging Science & Technology

**Introduction**

**Explain the process + informed consent**

**Warm up**

**-Introduce yourself:**

**-Where did you grow up?**

**-What kind of work/study do you do?**

**-A hobby.**

**Free Response & Associations**

**To explore initial perceptions of Synthetic Biology**

**-**What are the first things that come to mind when you hear '*Synthetic Biology*'?

**Introducing Synthetic Biology**

**Familiarize participants with an accessible definition and description**

**<<Present slide of short paragraph definition>>**

**<<Present 5-minute video on Synthetic Biology at CSIRO>>**

**-**What do you think about Synthetic Biology now that you've heard the description?

**-**Did it make you feel better, worse, or the same about it?

-any concerns?

**<<Probe for argument/justification>>**

**<<<**What do you mean by that?**>>>**

**<<Encourage to justify *why* a reference/story/image/metaphor was used>>**

**<<<**What is it about that reference/story/image/metaphor that resonates?**>>>**

**<<<**Why do you think it matters?**>>>**

**Vignettes**

**<<Applications (Select 2 per focus group)>>**

1. GD

2. Milk

*2.* Plastic

3. Cotton

**<<Discuss one at a time. Rotate across groups>>**

**<<Read first part of vignette for application>>**

**<<<Present the slide with the definition of each technology>>>**

**-Reiterate name of technology/application**

**-Ensure technical description is read and viewable**

**-**What do you think about this technology? Its intended purpose?

**-**Does this example make you feel better, worse, or the same about SynBio? In what way?

-any concerns?

**<<For each technology, probe for argument/justification>>**

**<<Encourage to justify *why* a reference/story/image/metaphor was used>>**

**<<<**What is it about that reference/story/image/metaphor that resonated?**>>>**

**<<<**Why do you think it matters?**>>>**

**<<Read remainder of the vignette, w/character disagreements and views>>**

**-**Did you agree w/anyone in this scenario? *Disagree with anyone?*

**-**What would you do? If you were in this group, would you try it/accept it?

***Vignette closer (the focus of this analysis)***

***Last thoughts on vignettes & a forced choice***

***-****If you had to choose one application of the 2 that is rolled out, which would you prefer? Why?*

***-****If you would not want any of them rolled out, why?*

***-what concerns you about the other application?***

**Closing Thoughts/Remarks**

**Last thoughts & one message**

***-***What kind of problems should we be trying to solve with science and technology like SynBio?

**-**What is one message you would like to take back to the people responsible for making the decisions about how Synthetic Biology technologies are developed in Australia?

**-**Given what you’ve heard about SynBio, does it serve the public good? *What would you actually like to see this technology do?*

**<<Each participant>>**

**<<<Challenge to justify why this message matters/phrased the way it was>>>**

**End of Focus Groups**

**<<Debrief>>**

**<<<Thank participants>>>**

**<<<Ethical statement and reminder of contact details>>>**

**Vignettes**

***Dairy***

Three friends are in a restaurant. They’re handed the dessert menu and told everything uses ‘cow milk, without the cow’. This doesn’t mean plant-based alternatives like almond milk; it means synthetic milk developed in the lab.

*They use something called ‘Precision Fermentation’. It’s similar to the process for making beer or bread, which uses yeast. However, instead of making alcohol the yeast is genetically reprogrammed to make proteins – the same proteins found in cow’s milk.*

*Cow DNA is synthesised and inserted into yeast, which forms the proteins. These proteins are then combined with minerals to make the building blocks for cow’s milk, only ‘without the cow’. This is argued to reduce our reliance on land and cattle.*

The three friends do not see eye-to-eye.

Mike’s curious but says it doesn’t sound appetizing. The server says it will taste the same as regular dairy so Mike’s willing to try it. Anita won’t try it because she has various allergies – she can’t be sure what she can and cannot eat and this is completely new. Patrick’s uncomfortable and wants to leave. “Synthetic milk?” he says. “Really?”.

Mike says they shouldn’t be afraid of trying new things.

But Patrick wants to know *why* they’re using this milk? The server explains that global protein demand is going to double by 2050. Our current approaches are unsustainable and won’t meet this demand. Synthetic dairy like this will reduce our reliance on intensive farming, lower pollution, and be good for animal welfare. And at the same time, it will still taste nice because it’s made with cow DNA and precision techniques. He also assures Anita that everything is lactose free.

They decide to stay and order desserts, but Patrick refuses to order. Mike tries his ice cream and he’s pleasantly surprised, while Anita says her cheesecake looks ok but still won’t try it because of food allergies and this is something new. She lets Patrick try it instead. Patrick spits it out and pushes it away, saying “Nope. It isn’t natural. What are we putting in our bodies?” Mike responds, “You eat fast food – that’s not healthy, right?”

But Anita agrees with Patrick, saying, “Who wants to eat something grown in a lab anyway?”. “I’m sure this is fine,” says Mike. “This was developed by scientists.”

***Plastic***

One day, three friends see a lot of plastic washed up on the beach. “It’s getting worse each year,” one of them says.

Sarah decides to tell them about her research. She’s a chemical-biologist and believes her research will create a breakthrough in recycling and have benefits for Australia’s environment.

*She’s been designing new-to-nature enzymes, which ‘eat’ plastic. The inspiration came from a natural enzyme that evolved in a Japanese rubbish dump, which was discovered to be ‘eating’ plastic. This process took about 80-100 years.*

*Her research accelerates the evolution of enzymes like this, and creates something that has never existed in nature before. The enzyme she’s developing will now ‘eat’ plastic in less than 1 hour. It breaks the plastic down to its core molecules, which can be reused to make new plastic products.* *This is said to enable infinite recycling.*

Sarah’s friends, Monica and Cal, have different views. Monica thinks it’s a good idea, but wonders if these enzymes will end up in the outside world and start breaking down all our plastic. “It could be like when a disease spreads,” she says. Sarah assures her this can’t happen: these enzymes are immobilized in the lab: they cannot escape. They are not living organisms. They degrade into harmless amino acids, which occur everywhere.

Meanwhile, Cal doesn’t like the sound of any of it. Cal says it would be better if we stopped altogether. He says we should change our harmful industrial practices and end plastics in the first place. As far as Cal is concerned, Sarah’s work sounds like a way to keep companies rich by continuing unsustainable practices. Sarah says Cal doesn’t have a solution. She says we can’t just stop everything we’re doing. We have to find ways to make our industrial practices cleaner and more sustainable. Monica still worries about what this might do outside the lab.

***Cotton***

Mabel is shopping for clothes for her daughter, Sophie. Sophie wants a pink dress. She thinks it’s beautiful and pleads with her mother to buy it. Mabel agrees. When the cashier scans the dress, she notices the label reads “Intelligently Designed Cotton”. Later at home, Mabel looks this up online and discovers the dress was made from a new process that has genetically modified the cotton in the lab to produce its own colour as it grows.

She learns that this was achieved by using a *pre-tested modular approach of adding colour genes into the cotton plant’s DNA, changing the fibre cells of the cotton from their natural white colour to pink*. *This will reduce the need to use toxic dyes.*

Mabel’s husband, Amon, is uncomfortable with genetic modification – he thinks companies will end up patenting genes and even entire genomes, and this sounds like a way for them to do it. He doesn’t trust the scientists because they have no idea how it’ll end up being used. But he’s willing to consider it with more information. Mabel thinks living organisms shouldn’t be genetically altered to this extent in a lab. It goes against her core beliefs. For Mabel, we should be more careful with God’s creations. She wants to return their daughter’s dress.

However, their daughter’s upset. She says her Mum promised. She’s been learning about genetic modification at school. Most of our cotton is genetically modified anyway, *plus* new things like this aren’t bad for the environment; they’re actually good for it. It was done to reduce our reliance on chemical dyes, which are toxic to the environment. Amon feels a bit better about this, but he’s undecided as he still thinks scientists aren’t honest about whatever new risks this could pose. Mabel refuses and intends to bring the dress back to the store.

***Gene drive for pest control***

Coen and Kylie are livestock farmers from the Northern Territory. They’re invited to a farmer’s organization meeting to hear about a public-private venture which wants to test a new form of invasive species control, focusing first on rabbits. Coen and Kylie struggle with farming costs and invasive pests add to their overheads, so they’re interested. The method is *gene drive.*

It is explained thus: *the genes of rabbits have been modified in the laboratory so they can only give birth to males. Using a genome editing tool, which is like a molecular pair of scissors, a specific part of the DNA is cut and the new instructions inserted. A small group of modified rabbits will be released to breed with the wild population. The trait of giving birth to males-only will spread through the population. As the proportion of males rises, this will gradually reduce the rabbit population, keeping their numbers low – without the use of toxic pesticides, baits, trapping or culling.*

The farmers are asked to be part of a pilot project in their region.

Coen is keen. However, Kylie isn’t at all happy.

Kylie studied biology at university and worries about the new genetic material in the rabbits passing into the environment. Coen doubts this would happen because the scientists reassured everyone during their presentation. Kylie says this will end up harming both the farm and the environment.

“But we already use pesticides, baits and animal traps,” says Coen. “So, this will actually help the environment.”

Kylie’s not convinced. “This is changing nature.”

“But the rabbits aren’t even from here – they’re invasive,” says Coen.

“I don’t like the idea of having the power to genetically eradicate a whole species,” she responds.

“That’s not possible,” replies Coen. “It’s just suppression.” Coen’s agitated: “It’s a better solution than our current approach,” he says.

“That’s what they always say,” responds Kylie. “Then watch what happens - How do you reverse it when it goes wrong?”.

**References**

Attride-Stirling, J. L. (2001). Thematic networks: An analytical tool for qualitative research. *Qualitative Research*, *1*(3), 385-405.

Corbin, J., & Strauss, A. (2015). *Basics of qualitative research: Techniques and procedures for developing grounded theory*. Sage Publications, Inc.

Levitt HM, Bambergm M, Creswell, JW, Frost, DM, Josselson, R & Suárez-Orozsco C (2018) Journal Article Reporting Standards for Qualitative Primary, Qualitative Meta-Analytic, and Mixed Methods Research in Psychology: The APA Publications and Communications Board Task Force Report. *American Psychologist* 73(1), 26-46.

Willig, C. (1999). *Applied discourse analysis: Social and psychological interventions*. Open University Press.
